# FPGA-based scanner and *SerialEM* server for 4D-STEM electron tomography

**DOI:** 10.1107/S2059798326009630

**Published:** 2026-09-23

**Authors:** Shahar Seifer, Michael Elbaum

**Affiliations:** a Chemical and Biological Physics Weizmann Institute of Science Rehovot7610001 Israel; National Center of Biotechnology, CSIC, Spain

**Keywords:** 4D-STEM tomography, CSTET, *SerialEM*, *SavvyScan*, shadow montage

## Abstract

Four-dimensional scanning transmission electron microscopy (4D-STEM) enables the acquisition of diffraction patterns at every probe position in a dense array. For imaging applications this approach offers significant benefits in terms of spatial resolution and contrast enhancement. In this work, we present an update on our open *SavvyScan* platform, consisting of a synchronous scan generator integrated by software with *SerialEM* to enable the automation of complex experimental protocols such as tomography. The proposed hardware functions as an interface between *SerialEM*, the scan controls of the microscope, a fast annular dark-field detector and a synchronized trigger for a pixelated detector. Our previous implementation relied on a dedicated computer equipped with a multichannel acquisition and signal-generation cards, as well as a separate microcontroller for synchronization. Here, we report a low-cost implementation based on a Red Pitaya board, utilizing direct programming of its embedded FPGA and Linux server components. We provide detailed instructions for system installation and operation, along with practical guidance for modifying the source code. System performance is validated through oscilloscope measurements and imaging of a replica grating sample. The utility of the approach is further demonstrated by generating a 3D electron tomogram of a cryogenic sample of mitochondria from a tilt series of shadow montage projections.

## Abstract 2

Software for a multipurpose hardware board enables flexible scanning with a ready acquisition and processing pipeline to implement 4D-STEM electron tomography.

## 1. Introduction

This work is part of our long-standing endeavor to advance the modality of scanning TEM (STEM) tomography for life-science cryogenic samples (Wolf *et al.*, 2014 ▸). Cryo-scanning transmission electron tomography (cryo-STET) enables three-dimensional imaging of unstained, fully hydrated vitrified cells, in which flexible modes of STEM signal formation alleviate the thickness limitation inherent to conventional energy-filtered cryo-TEM. This provides access to larger cellular volumes with improved depth resolution, particularly at high tilt angles, while preserving native-state ultrastructure.

We consider the use of the *SerialEM* software (Mastronarde, 2003 ▸) to be central to the workflow, as described in Kirchweger *et al.* (2023 ▸). The workflow supports low-electron-dose searching for features of interest, registration of target areas and tracking. It also enables automated tilt-series acquisition for tomography by controlling sample tilting and tracking to stabilize the field of view. *SerialEM* is compatible with electron microscopes from all major manufacturers and can connect to multiple camera servers, including STEM systems that generate images using a scanning probe. The Camera Setup interface in *SerialEM* allows users to define scan parameters such as dwell time, image size and pixel sampling density. It further supports dynamic focusing in STEM by adjusting the microscope optics according to the beam position on a tilted sample. The hardware interface to a camera operates via a server model. Our original *SavvyScan* system (Seifer *et al.*, 2021 ▸; Seifer & Elbaum, 2023 ▸) is one such server, providing STEM images based on a selected detector. It incorporates an independent scan engine that connects to the *X* and *Y* channels of the microscope beam deflectors. The server integrates into *SerialEM* via a plugin, while operating as an independent process that stores additional datasets acquired simultaneously from multiple detectors (up to eight channels). Accordingly, the output from an azimuthally segmented quadrant detector is used for integrated differential phase-contrast (iDPC) techniques (Seifer *et al.*, 2021 ▸; Kirchweger *et al.*, 2025 ▸), while recordings from a pixelated detector enable 4D-STEM techniques (Seifer, Kirchweger *et al.*, 2024 ▸; Seifer, Houben *et al.*, 2026 ▸).

Recent developments in 4D-STEM have shown clear practical advantages in high-resolution imaging and analysis (Küçükoğlu *et al.*, 2024 ▸; Yu *et al.*, 2025 ▸; Shi *et al.*, 2022 ▸). By acquiring a full diffraction pattern at each probe position, the method captures more complete scattering information than conventional STEM, which can then be reconstructed in different modes from the same dataset. This enables improved contrast, particularly for weakly scattering and beam-sensitive samples. In addition, the use of phase-retrieval methods such as ptychography can enhance spatial resolution beyond that of conventional STEM techniques (Küçükoğlu *et al.*, 2024 ▸; You *et al.*, 2025 ▸). A variant of such approaches is our shadow montage technique that resolves layers in the sample by summation of diffraction patterns treated as cone-beam shadow projections (Seifer, Houben *et al.*, 2026 ▸). A different analysis of similar raw data is used for the tilt-corrected bright field (tcBF) method based on parallax correction (Yu *et al.*, 2025 ▸). Furthermore, because the recorded signal retains angular scattering information, it can be analyzed quantitatively to provide material characteristics based on inferred elastic and inelastic scattering contributions (Seifer, Houben *et al.*, 2024 ▸). Our aim has been to extend these capabilities to three dimensions by supporting tilt-series acquisition in 4D-STEM. Here, we present *SavvyScan* in a second-generation hardware platform dedicated to 4D-STEM. It is at once flexible, inexpensive and designed from the outset with *SerialEM* integration in mind. Based on a Red Pitaya development board, ironically named STEMlab, it is also compact, portable and especially simple to implement.

## 2. System design

**Figure 1:**
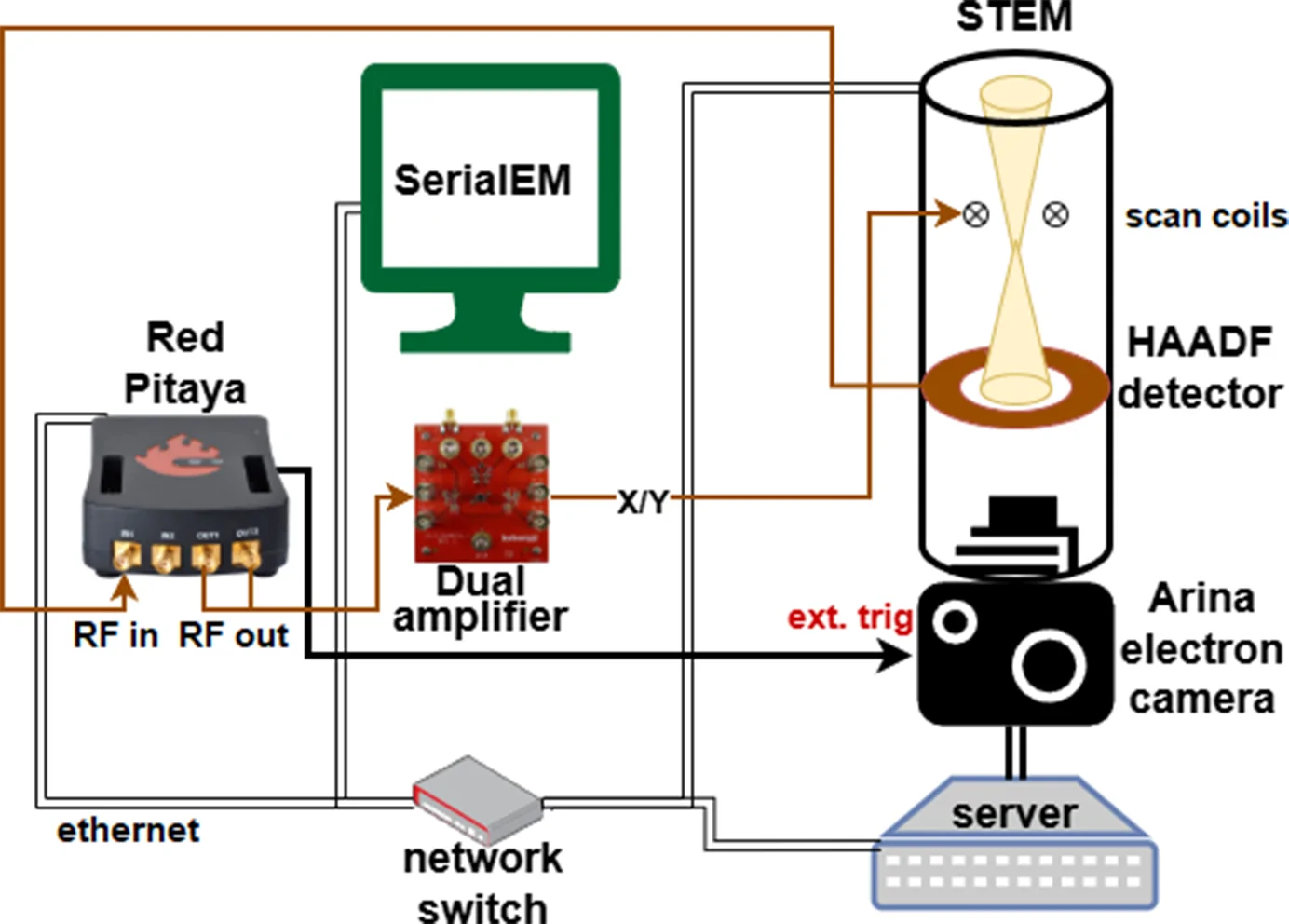
Scheme of the Red Pitaya FPGA-based *SavvyScan* system and connections to the STEM microscope.

The original *SavvyScan* system has been described in our previous publication (Seifer *et al.*, 2021 ▸; Seifer & Elbaum, 2023 ▸), and we focus here on design changes and the motivation. Table 1 ▸ summarizes differences in capabilities between the Red Pitaya FPGA design and the previous platform based on Spectrum Instruments cards, which include a multichannel analog-to-digital converter (ADC) and arbitrary wavefunction generator (AWG). The new system diagram is shown in Fig. 1 ▸, and its technical implementations are discussed in Appendix *A*[App appa]. The Red Pitaya board is connected via a local ethernet network to a Windows workstation running *SerialEM* and to a 4D-STEM camera server. In our system the latter is the DECTRIS server unit for controlling an ARINA pixelated detector via HTTP-REST commands. An ethernet connection between *SerialEM* and the electron microscope is also required, optionally over a separate network. Two analog outputs of the Red Pitaya board are connected to the microscope scan controls, and a digital output serves to trigger the pixelated detector. As a scan engine, our software assumes a certain scan polarity according to that available in Tecnai F20 (FEI Inc). In the Titan Krios (Thermo Fisher Scientific), by contrast, the polarity is selected by a choice of socket. The system requires that an interface for an external scan generator is available in the microscope and that the pixel calibration is consistent between magnifications.

*SavvyScan* is designed with flexibility as a core principle, most notably through programmable scan patterns. It was originally intended for simultaneous acquisition from multiple detectors, such as the Opal segmented diode (El Mul Technologies), and later adapted for 4D-STEM (Seifer & Elbaum, 2023 ▸). In the new FPGA implementation, we retain the same data structures as in the previous version. For a conventional raster, we define a scan pattern that begins outside the imaging area so that the scanning probe reaches steady velocity before entering the region of interest. Notably, trigger patterning provides a synchronized stream of trigger pulses only in the image area, avoiding the need to clean the data files later. The scan pattern is stored in on-board RAM and executed directly on the FPGA, providing for additional scan patterns such as zigzag, spiral, sliding circles *etc.*

In contrast to the previous design, which supported eight ADC channels, the current implementation uses a single ADC channel for sending images to *SerialEM*. We conveniently use the high-angle annular dark-field (HAADF) detector to provide the housekeeping signals for navigation, tracking, focus *etc.* Diffraction images are acquired on a pixelated detector at selected scan positions defined by a gate pattern table. (The same feature might be used to control a fast electrostatic shutter for compressive sensing applications.) Diffraction data are recorded directly on the detector server without passing back to *SerialEM*. In the new register-transfer-level implementation there is no lower bound for the scan rate, and a new feature enables a variable trigger delay to support frozen beam acquisition at each scan point.

The new design simplifies user operation by optionally eliminating a separate user interface and reducing configuration elements. The goal is to provide a transparent workflow that can be operated in a manner similar to conventional electron tomography, without requiring extensive training in 4D-STEM techniques. All system operations are handled directly from *SerialEM*, and dedicated 4D-STEM control is provided through *SerialEM* script functions.

The overall architecture is simplified through direct FPGA programming, which removes several constraints present in the earlier implementation. Over-sampling and averaging of the HAADF ADC signal within a single pixel dwell time, for noise reduction, is now performed directly on the FPGA. This significantly reduces the processing load on the server. Such an approach has shown an improved signal-to-noise ratio in other scanning-probe systems (Gong *et al.*, 2026 ▸). A limitation of the current implementation is the available on-board memory of 256 MB, which currently restricts the maximum scan size to 2048 × 2048 points. In practice this constraint is not limiting for typical applications, especially in light of the up-sampling made possible by defocused 4D-STEM methods such as ptychography, tcBF and shadow montage.

Typical camera settings in *SerialEM* include *Search* (quarter FOV, 256 × 256, 30 µs per pixel), *Trial* (full FOV, binned to 1024 × 1024, 6 µs per pixel) and *Focus* (quarter FOV, 512 × 512, 1 s per frame). In *Record* the FOV is full, binned to 1024 × 1024 or 2048 × 2048. Note that the flyback time is included in the *SerialEM* display of the pixel dwell time. The ARINA camera reaches 8 µs frame speed only when operating in free-running mode. Using the external trigger to avoid acquisition during flyback and acceleration, we recommend a 14 µs trigger interval for stable operation, which requires 20 µs dwell in the Camera Setup due to the extra flyback time.

A script file contains available actions that are not implemented in *SerialEM* but can be run from its script engine via CallFunction send_command <property>. The single-line calls are deactivated by comment characters (#) and include the properties listed in Table 2 ▸. Additional options related to communication with the ARINA are under development.

A tilt series in 4D-STEM may be started by the ARINA and StartTiltSeries script commands with a unique filename. Typically, we use the eucentric height calibration and tilt-series acquisition available in the *SerialEM* menu and select a dose-symmetric tilt order. For maximum stability, particularly with a side-entry holder, select ‘Tracking by Trial scans only’. Different protocols for single-particle analysis or micro-ED/3DED may be adopted straightforwardly by means of *Serial­EM* scripting (Jones *et al.*, 2018 ▸; Nexperion, 2026 ▸). Our GitHub repository contains *SerialEM* scripts for hands-free batch acquisition of 4D-STEM tilt-series at selected positions using the Navigator functions, including Anchor Maps and all available tools for automation. The HAADF output is saved to an MRC file, whereas the 4D-STEM dataset is stored via the DECTRIS server filewriter. (Interface to cameras of other suppliers should be straightforward via modification of the HTTP calls, although this has not been tested.) To facilitate orderly recording of metadata such as tilt-series parameters, scan, detector and microscope settings we provide a GUI application called Session-JSON, also available on GitHub.

## 3. Results

**Figure 2:**
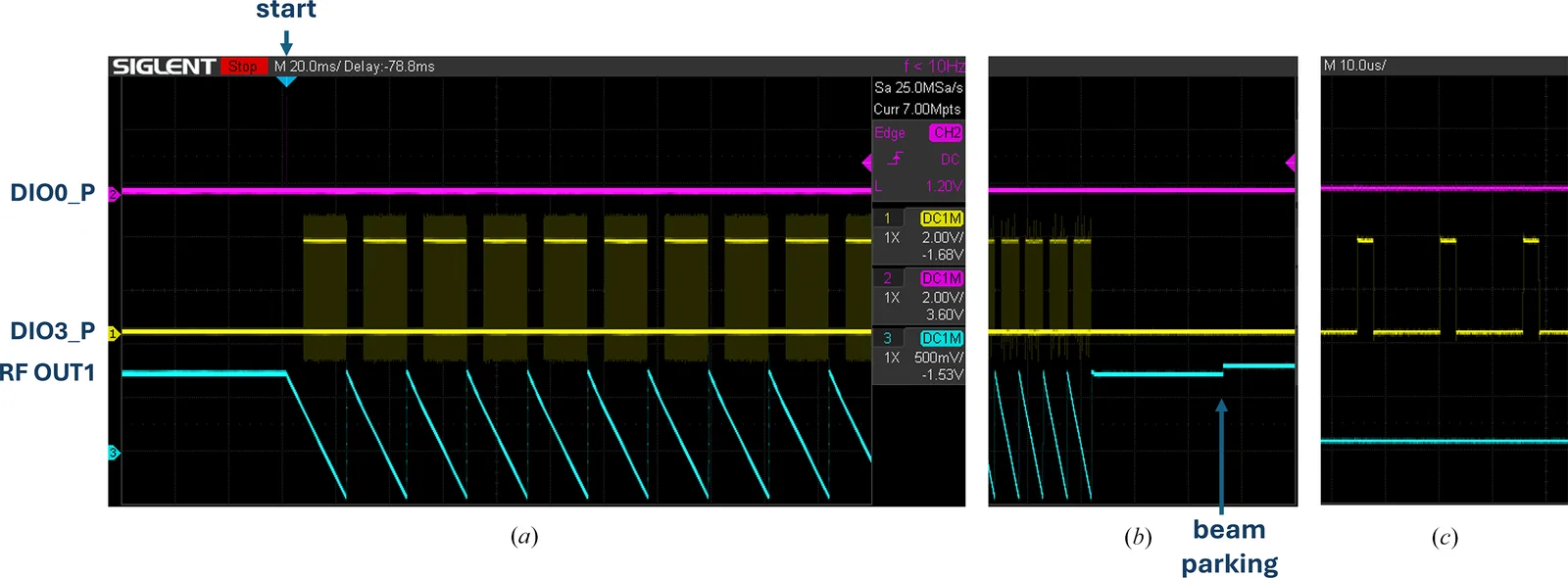
Signals on a scope demonstrate synchronization during a scan. DIO0_P serves internally to start acquisition. DIO3_P is the trigger for the pixelated detector (ARINA) based on 1024 pulses in each line of the scan. RF OUT1 is driving the *X* scan coil of the microscope. Trace (*b*) shows the end of the scan in which the signals are synchronized and the beam-parking state is engaged. Trace (*c*) shows the pattern of single trigger pulses.

The oscilloscope trace shown in Fig. 2 ▸ validates FPGA operation with precise synchronization between the analog output (RF OUT1) and the camera trigger signal (DIO3_P). The oscilloscope is triggered by the DIO0_P output, which is generated by the FPGA upon receiving a command cue in RAM to initiate the scan. The DIO3_P pulses are 3 µs wide and repeat for each pixel of the generated 1024 × 1024 HAADF image broadcast to *SerialEM*. Each burst of pulses corresponds to the acquisition of a single image line, where the final camera trigger ends immediately before the scan *x* voltage returns to a position outside the field of view in preparation for the next scan line. This synchronization is maintained throughout the entire scan. At the conclusion of the scan, the beam is deflected to a distant parking position to avoid sample damage within the field of view. An Arduino microcontroller was used to confirm that exactly 1024 × 1024 trigger pulses are received.

**Figure 3:**
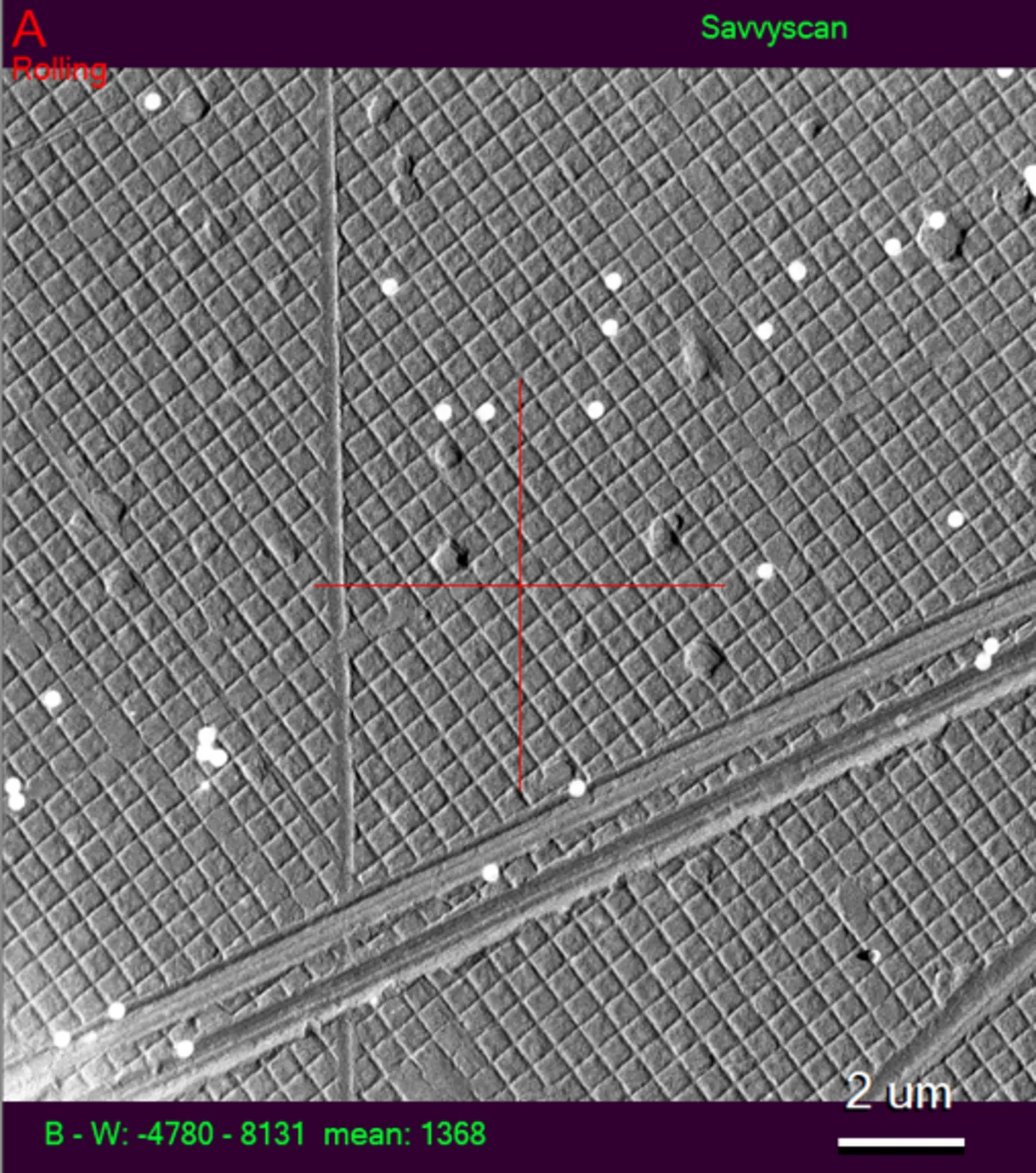
The output window in *SerialEM* after scanning of a standard replica grating. The image demonstrates geometric uniformity over the field of view, and a proper calibration based on the specified grating density of 2160 lines per millimetre.

To test synchronization between the output and input channels one can immediately verify the correct imaging pattern by directly connecting one output channel to the input CH1. For a complete validation, we tested a standard replica grating sample (EMS) on a Tecnai-F20 S/TEM microscope (FEI Inc.) operating at 200 kV acceleration in NanoProbe mode with a 30 µm C2 aperture (4 mrad semi-convergence angle) and a HAADF detector (Fischione). The result shown in Fig. 3 ▸ demonstrates straight grating lines that verify correct geometrical mapping and linearity of the dual amplifier (configured as an inverting operational amplifier with a gain of −11), and the spatial calibration is consistent with the specified grating density of 2160 lines mm^−1^. Clear contrast is observed between the carbon layer (gray) and the latex bead (white). Correct calibration is also validated by shifting the scan position and confirming the agreement between the predicted and measured locations.

**Figure 4:**
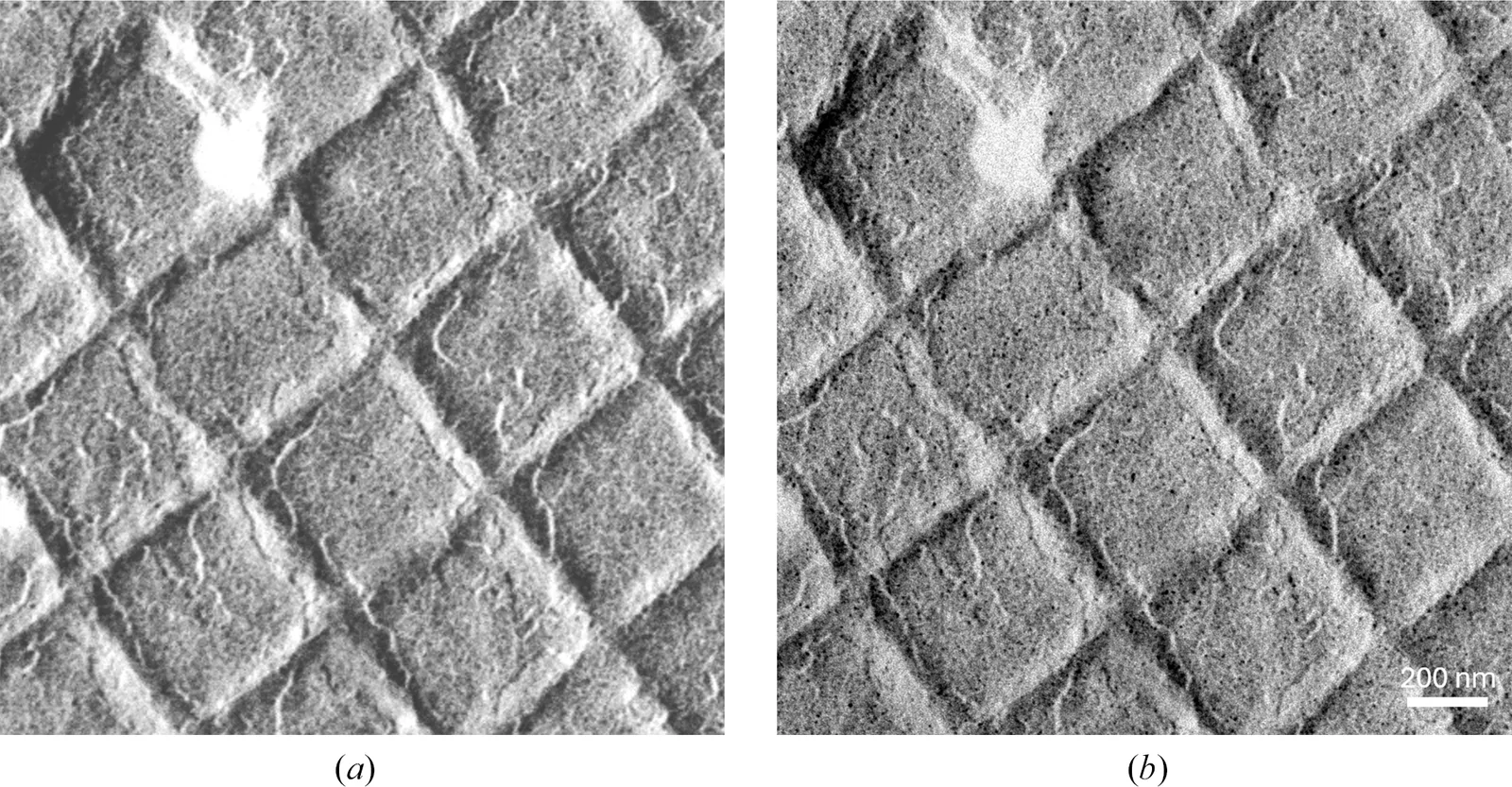
Magnified scan of a replica grating sample with 512 × 512 probe positions. (*a*) HAADF image, inverted in contrast. (*b*) Shadow montage based on the 4D-STEM dataset demonstrating upscaled resolution (note the Au/Pd nanoparticles as black dots).

At higher magnification, zooming into the center of the low-magnification image is demonstrated. The HAADF image shown in Fig. 4 ▸(*a*) was acquired using a 512 × 512 scan. Simultaneously, a 4D-STEM dataset was collected using the ARINA pixelated detector, comprising 512 × 512 diffraction images of 96 × 96 pixels each. Applying the shadow montage technique to this dataset yields the image shown in Fig. 4 ▸(*b*), which exhibits improved resolution and contrast. Au/Pd nanoparticles present on the commercial sample are clearly resolved as dark features.

**Figure 5:**
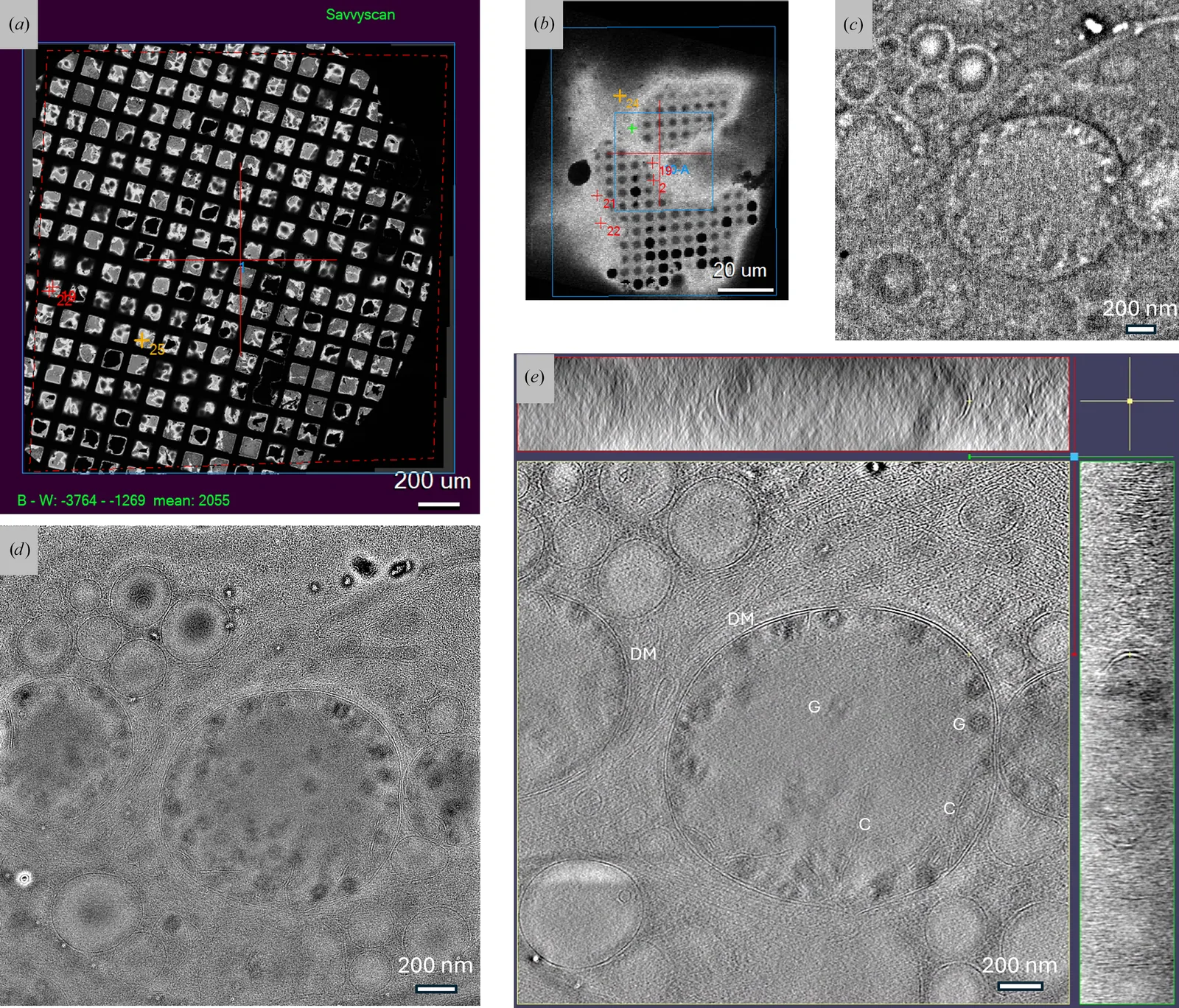
Electron tomography of mitochondria in a U2OS cell sample using the updated *SavvyScan* and post-processing by the shadow montage technique. (*a*) Overview of the grid generated by *SerialEM* from a montage of low-magnification HAADF scans. (*b*) One grid-square scan controlled by *SerialEM*. (*c*) Direct HAADF image of one projection. (*d*) Post-processing a shadow montage based on the 4D-STEM dataset of one tilt view. (*e*) A tomogram built from 37 tilt views in shadow montage (labels: DM, double membrane; G, calcium phosphate granule; C, cristae).

The usefulness of the system is demonstrated using a cryo-sample of U2OS cells grown on a gold-coated Quantifoil grid. The Tecnai-F20 microscope was set to MicroProbe mode with a 70 µm C2 aperture, resulting in a beam semi-convergence of α = 1.9 mrad. Using *SerialEM* with the HAADF detector, an overview mapping of the grid is shown in Fig. 5 ▸(*a*), a square map in Fig. 5 ▸(*b*) and a scan at the region of interest in Fig. 5 ▸(*c*). The latter is based on 1024 × 1024 scan points with step size *d* = 3 nm per pixel. The probe was intentionally overfocused by Δ*f* = 10 µm to support the formation of shadow images in the diffraction plane. The defocus remained stable throughout the tilt views by activating dynamic focusing in the *SerialEM* camera setting. The bright-field disk illuminating the pixelated detector covered a diameter of *N*_BF_ = 53 pixels. A 4D-STEM dataset was acquired for a tilt series in dose-symmetric tilt-view order, with angles starting at 0, −3, −6, −9, 3, 6, 9 and so forth, covering the range between −54 and 54°. The dataset was processed according to the shadow montage technique with a synchronization value of *N*_S_ = 4 according to the formula *N*_S_ = (*N*_BF_*d*)/2αΔ*f* (Seifer, Houben *et al.*, 2026 ▸). The ShadowMontage_tiltseries_ver2.m script (Seifer, 2026 ▸) applies a CTF correction for spatial frequencies between the highest represented by the diffraction-limited probe and that of the illuminated patch. Normal incoherent bright-field contrast is retained for lower frequencies, providing consistent contrast across scales. The 4D-STEM dataset and resulting aligned shadow montage tilt series is shared in Zenodo (Seifer, Dalaloyan *et al.*, 2026 ▸). One tilt-view example is shown in Fig. 5 ▸(*d*), clearly resolving details such as double membranes, cristae and calcium phosphate granules in three nearby mitochondria (Wolf *et al.*, 2017 ▸). The 3D reconstruction shown in Fig. 5 ▸(*e*) was generated by alignment using *IMOD* (Kremer *et al.*, 1996 ▸) and a subsequent SIRT reconstruction using *Tomo*3*d* (Agulleiro & Fernandez, 2015 ▸). 3D traces of the cristae, granules and vesicles are easily observed in the two side-projection views.

## 4. Conclusions

We have presented an updated *SavvyScan* based on a general-purpose development board with fully open-source FPGA firmware and C++ code. The system provides practical and automated acquisition of 4D-STEM data in *SerialEM*, and is demonstrated for automated tomography. After installation the operation should be nearly transparent to *SerialEM* users. The hardware is available off the shelf and is affordable for any laboratory, offering further customization for particular needs. The modular, open software suggest its adaptability to other protocols supported in *SerialEM*, such as MicroED, as well as to other hardware or to SEM-based tools requiring a custom scan and control system.

## Supplementary Material

Link to software.: https://github.com/Pr4Et/SavvyScan/tree/main/Savvyscan%20on%20Red%20Pitaya

Raw 4D-STEM tilt-series data of a cryoEM sample.: https://doi.org/10.5281/zenodo.20544863

## Figures and Tables

**Table 1:** Comparison between *SavvyScan* platforms

	Spectrum Instruments ADC/AWG-based *SavvyScan*	Red Pitaya FPGA-based *SavvyScan*
Footprint	Desktop PC, 8-channel ADC, 2-channel AWG, STAR hub, MCU board, 18 cables	Red Pitaya card, amplifier card, 2 power supplies, 8 cables
4D-STEM tomography supported	Yes (version 2)	Yes
Segmented detectors supported	Yes	No
Sampling rate (analog input/output)	Min: 128 kS s^−1^	Min: 0.1 S s^−1^
	Max: 5/20/40/80/125 MS s^−1^ (model dependent)	Max: 125 MS s^−1^
Sampling resolution (analog input/output)	16-bit	14-bit
Scanning-rate bottleneck	Signal integration	Image transfer
Scan size	Unlimited (with FIFO buffer)	Limited by onboard memory
Synchronization and robustness	8-sample synchronization misalignment between STEM and 4D-STEM. External clock reference. Restricted selection of timing parameters.	Synchronization aligned. Internal synchronization with robust performance according to FPGA specification. Controlled trigger delay allows frozen beam acquisition.
Pattern options	Implemented: raster, spiral, sliding circle, Lissajous	Programmable in C++ code (feeder.cpp)
Fully manageable from *SerialEM*	No	Yes
Cost	>15 000 USD (model dependent)	∼600 USD

**Table 2:** Properties of the function send_command in *SerialEM* scripting

Property	Description
ARINA: <filename>	Arm the ARINA camera and set a master filename
StartTiltSeries: <number>	Start a tilt series, and if ARINA is armed keep this state for the specified number of tilt views and write their counter in the hdf5 filename
StopTiltSeries	End the tilt series prematurely
SetThTimeus: <time µs>	Record 4D-STEM datasets for scan requests longer than this threshold per scan position (default 13 µs)
SetTriggerDelayus:<time µs>	Set a delay between the signal to the scan coils and a trigger to the pixelated detector (default 1 µs)
EnergyKV:<energy keV>	Report the electron energy to the pixelated detector
ScanPattern:<number>	1 − raster scan (default)

## Data Availability

The source code and installation files are available at https://github.com/Pr4Et/SavvyScan/tree/main/Savvyscan on Red Pitaya.

## Appendix A Installation and operation

### A1. Technical implementation

The present system is implemented on a Red Pitaya STEM 125-14 Gen-I board, which is based on a Xilinx Zynq-7010 FPGA, with dual-channel ADC and AWG, both with 14-bit resolution, along with 256 MB of DDR memory. It also includes an integrated Linux development environment, which we use as a server to *SerialEM*. Adaptations for the Red Pitaya STEM 125-14 Pro Gen-II board, based on the Zynq-7020 FPGA and equipped with 512 MB of memory, are also planned and will be made available in our repository. The external trigger output is available on the DIO3_P pin of an extension connector of the board.

Communication between the software stack and the FPGA is handled via several paths. The system bus RAM is used to store control queues and parameters. BRAM is employed for transferring gate patterns, similarly to our previous *SavvyGate* system. DMA with a FIFO buffer is used to transfer AWG patterns from the software to DDR RAM, and subsequently from DDR RAM to the FPGA. Analog signal acquisition and storage in DDR RAM are based on an FPGA implementation provided by Red Pitaya. Data acquisition is triggered by a pulse on the DIO0_P pin, which can be physically monitored. Further details of the FPGA design are available in the Vivado project that we share on GitHub. All these details are transparent to the user.

To drive the scan controller over its full allowable range (higher than ±1 V), the system incorporates an external dual-channel JFET operational amplifier (ISL28210S, Intersil). The amplifier is selected with high slew rate (above 20 V µs^−1^), 10 MHz gain-bandwidth and 100 dB common mode rejection. These are required to prevent instability by reflections and to support linear response (resistive termination must be avoided). An inverting gain configuration is implemented by soldering SMT resistors on the board according to the Instersil ISL28210SOICEVAL1Z evaluation board user’s guide (Application Note 1594). For a gain of −11 the parameter output_amplifiers_gain_divider in the file feeder.cpp was set to 4.4 for the Krios and 6.5 for F20, in a particular *SerialEM* calibration. A regulated power supply of ±12 V/0.5 A is suitable.

### A2. Build instructions

The instructions below are intended for a specific Red Pitaya board (RPB) STEM 125-14 gen1 connected via a local Ethernet switch to a Windows personal computer (WPC). The internet protocol (IP) name of RPB is denoted by rp-xxxxxx.local.

#### A2.1. SD card preparation

Prepare the SD card according to the instructions in Section 2.5: Prepare SD card — Red Pitaya Documentation with software version (2.07-48).

#### A2.2. Setting IP addresses

Use a web browser to establish the initial connection. Type rp-xxxxxx.local and select ‘Development’ and ‘Web Console’ in the graphical user interface. The RPB IP address may be read by command

ip addr

An address that complies with the local microscope network can be set as follows:

nano /etc/systemd/network/ 20-wired-static-eth0.network

Enter the following content:

[Match]

Name=eth0

[Network]

Address=192.168.100.90/16

Save the file by Ctrl+O and Ctrl+X. Activate the configuration by running

systemctl restart systemd-networkd.service

The IP address and mask of the WPC ethernet socket should be set appropriately. For example, in Ethernet settings change the TCP/IP properties to

IP: 192.168.100.50

Subnet mask: 255.255.255.0

The DECTRIS server address should be set accordingly to the content of the start script file:

IP: 192.168.100.70

Subnet mask: 255.255.255.0

#### A2.3. SSH connection

To connect with RPB Linux environment, establish SSH authentication between WPC and RPB. Install OpenSSH on the WPC and use PowerShell commands:

ssh-keygen -t rsa -b 4096 (press Enter 3 times).

type $env:USERPROFILE\.ssh\id_rsa.pub | ssh root@rp-xxxxxx.local "mkdir -p ~/.ssh && cat >> ~/.ssh/authorized_keys" (then answer ‘root’ for password).

The authentication is needed once per WPC. To connect with another PC, repeat the above steps, leaving out the part "mkdir -p ~/.ssh && ". The command in PowerShell is

ssh root@rp-xxxxxx.local

To copy files from WPC to RPB exit the RPB environment and use

scp [Windows file] root@rp-xxxxxx.local:~/[Linux folder]/

#### A2.4. Optional preparation of a new FPGA binary

Download Vivado 2025.2 full version (not lab version), which can be installed with the free Vivado WINPACK license. Download the project from our Github repository and open prj/v0.94/project/redpitaya.xpr. The relevant files are red_pitaya_top.sv, red_pitaya_ps.sv, system.bd and rp_gate_sys_extmem.sv. Double click the system.bd icon in sources explorer to open the system block scheme, which is the hardware component that contains the processing system and our modifications. To compile the changes follow these steps: Validate Design, Generate Output Products, Run Synthesis, Run Implementation, Generate Bitstream. Copy red_pitaya_top.bif to folder prj\v0.94\project\redpitaya.runs\impl_1, then in the TCL console run

cd <RedPitaya-FPGA folder>/prj/v0.94/project/redpitaya.runs/impl_1

bootgen -image red_pitaya_top.bif -arch zynq -process_bitstream bin -w

This will generate a new FPGA binary red_pitaya_top.bit.bin instead of the one we provide.

#### A2.5. Install the FPGA binary stack

Copy the binary FPGA file red_pitaya_top.bit.bin to the RPB home folder (~ or /root). On the RPB (Linux), run the following commands from the /root directory:

cp red_pitaya_top.bit.bin /opt/red_pitaya_top/fpga.bit.bin

/opt/redpitaya/sbin/overlay.sh red_pitaya_top × y Full

Check the printed status to confirm that the FPGA is running.

#### A2.6. Install the C++ *SerialEM* server software

In RPB Linux make the directories ~/Savvyscan­Pitaya/src, ~/SavvyscanPitaya/include and /opt/velan/Lib. Copy to the RPB home folder (~ or /root) the bash-script files compile and start (related to compiling and running the *SerialEM* server on RPB). Copy the C++ source files and headers to the *SavvyScan*src and include folders. Copy the Python library content to /opt/velan/Lib.

Download and install *tifffile* for the Python environment:

pip3 install tifffile-2024.9.20-py3-none-any.whl

Compile the C++ code by running in Linux from the /root folder:

bash ./compile

#### A2.7. *SerialEM* installation

On a Windows PC intended for *SerialEM*: download and run a 64-bit *SerialEM* package from SerialEM Downloads and Installation. A subfolder under Program Files\Serial­EM should be installed.

Allow writing permissions for the *SerialEM* folder, start a command terminal or PowerShell and cd to the *SerialEM* installation subfolder. Use

run install.bat (and answer to questions related to your specific microscope).

Copy the plugin file savvyscan.dll to the *SerialEM* folder.

A Windows environment variable SAVVY_SERVER_IP should be set equal to the RPB IP address (in Windows, go to Advanced System Settings, Environment Variables and add this user variable). The default address is 192.168.100.90, which overrides the address written in the SerialEM­properties file.

Create a configuration folder Start_SerialEM which should contain the files SerialEMsettings.txt, SerialEMproperties.txt, SerialEMsettings-scripts.txt and SEMsystemSettings.txt, which can be copied from our repository. The SerialEM­Settings files must be modified in the first lines according to the actual folders, for example

SystemPath <Start_SerialEM folder>

ScriptPackagePath <Start_SerialEM folder>\SerialEMsettings-scripts.txt

The SerialEMproperties.txt file should be modified according to the actual folders in use and according to the microscope IP address:

SocketServerIP 1 192.168.100.30 #microscope PC address

SocketServerPort 1 48892 #fixed port number

Add a shortcut in folder Start_SerialEM and edit its properties: Target should specify the full path to SerialEM.exe in Program Files and Start in should specify the full path to the Start_SerialEM folder.

#### A2.8. *SerialEM* calibration

Follow the instructions in the *SerialEM* documentation for proper calibration of the pixel size, stage and image shifts. If the pixel calibration has already been made for a different *SerialEM* camera the scan voltage range may be adapted by changing the related parameters in feeder.cpp

(output_amplifiers_gain_divider and parking_voltage)

and compiling the code

bash ./compile

Ensure that the image is not distorted at the margins, which is caused by clipping at either the RPB output (±1 V) or the input interface of the microscope control. On our Krios microscope we avoid using microprobe magnifications below ×5000 because these are not consistent with higher magnifications.

### A3. Operating instructions

Power up the Red Pitaya Board (RPB) and the dual amplifier.

Start a command terminal and SSH into the RPB Linux server. Start the server by typing in the /root folder:

bash ./start

Run *SerialEM* using the shortcut in the Start_Serial­EM folder.

Single images are acquired as normal in *SerialEM* using the Camera Setup and optionally the Low Dose controls. Make sure that the HAADF detector is inserted and selected in the Camera Setup window, and adjust the HAADF contrast/brightness to the proper range. The full digital range is supported by *SerialEM* in camera option ‘Divide 16-bit by 2’. Ensure that the external scan is enabled in order to bypass the internal scan generator.
